# Stochastic Effects in Retrotransposon Dynamics Revealed by Modeling under Competition for Cellular Resources

**DOI:** 10.3390/life11111209

**Published:** 2021-11-09

**Authors:** Sergey Pavlov, Vitaly V. Gursky, Maria Samsonova, Alexander Kanapin, Anastasia Samsonova

**Affiliations:** 1 Mathematical Biology & Bioinformatics Laboratory, Peter the Great Saint Petersburg Polytechnic University, Saint Petersburg 195251, Russia; sergpav92@mail.ru (S.P.); m.samsonova@spbstu.ru (M.S.); a.kanapin@gmail.com (A.K.); 2 Theoretical Department, Ioffe Institute, Saint Petersburg 194021, Russia; 3 Theodosius Dobzhansky Center for Genome Bioinformatics, Saint Petersburg State University, Saint Petersburg 199034, Russia

**Keywords:** mobile genetic elements, retrotransposons, cellular resources, predator–prey model, stochastic dynamics, Gillespie algorithm

## Abstract

Transposons are genomic elements that can relocate within a host genome using a ‘cut’- or ‘copy-and-paste’ mechanism. They make up a significant part of many genomes, serve as a driving force for genome evolution, and are linked with Mendelian diseases and cancers. Interactions between two specific retrotransposon types, autonomous (e.g., LINE1/L1) and nonautonomous (e.g., Alu), may lead to fluctuations in the number of these transposons in the genome over multiple cell generations. We developed and examined a simple model of retrotransposon dynamics under conditions where transposon replication machinery competed for cellular resources: namely, free ribosomes and available energy (i.e., ATP molecules). Such competition is likely to occur in stress conditions that a malfunctioning cell may experience as a result of a malignant transformation. The modeling revealed that the number of actively replicating LINE1 and Alu elements in a cell decreases with the increasing competition for resources; however, stochastic effects interfere with this simple trend. We stochastically simulated the transposon dynamics in a cell population and showed that the population splits into pools with drastically different transposon behaviors. The early extinction of active Alu elements resulted in a larger number of LINE1 copies occurring in the first pool, as there was no competition between the two types of transposons in this pool. In the other pool, the competition process remained and the number of L1 copies was kept small. As the level of available resources reached a critical value, both types of dynamics demonstrated an increase in noise levels, and both the period and the amplitude of predator–prey oscillations rose in one of the cell pools. We hypothesized that the presented dynamical effects associated with the impact of the competition for cellular resources inflicted on the dynamics of retrotransposable elements could be used as a characteristic feature to assess a cell state, or to control the transposon activity.

## 1. Introduction

The ability of mobile genetic elements (transposons or jumping genes) to move within a host genome makes them a powerful instrument to study and trace evolutionary changes [1,2,3]. Approximately half of the human genome consists of transposable elements, which were active during different periods of human evolution [4,5]. Apart from the adaptive function utilized millions of years ago, the site-to-site movements of retroelements within a host genome may perturb its integrity and lead to various genetic disorders associated with a plethora of diseases [6,7]. To uncover the mechanisms involved in the control of genomic rearrangements caused by the activity of transposons, it is important to understand the dynamical consequences of their relocations and their interactions with the cellular environment.

There are two major classes of transposons, which differ by the mechanism used to move within the host genome. DNA transposons utilize the ‘cut-and-paste’ mechanism, excising themselves from the genome and inserting themselves into a different location [8]. Retrotransposons can copy themselves into new genomic locations, keeping their source sequences and thus exploiting the ‘copy-and-paste’ mechanism [2,9]. DNA transposon copies currently present in the human genome are inactive, thus presenting fixed evidence of their activity during the early primate evolution. Conversely, retrotransposable elements are currently mobile in humans and are therefore of particular interest [4,5,10]. Thus, the type 1 long interspersed element (LINE-1, or L1) provides an excellent example of the autonomous class, accounting for approximately 17% of the human genome [4,11]. Another difference between the two types of mobile elements is the way in which transposons utilize cellular molecular machinery for integration into the genome. Autonomous transposons encode mRNA and the proteins needed for their transposition. Non-autonomous transposons do not encode their own molecular machinery required for the integration into the genome, but rather utilize the proteins encoded by the autonomous ones. A typical example of non-autonomous retrotransposons is the Alu element, which belongs to a class of the so-called short interspersed elements (SINEs) [12,13,14]. Alu, together with another non-autonomous retrotransposable element—SVA—constitute about one-third of the human genome [2,4]. 

The dynamics of transposons in the genome is an attractive subject for mathematical modeling. One approach uses methods of population genetics to model the neutral evolution and equilibrium distribution of transposons in a population [15,16]. In bacterial genomes, the neutral evolution model adequately explains the dynamics of transposable element abundance over large evolutionary scales, as transposons and their hosts tend to maintain a dynamic equilibrium [17]. Other models use the genomic data and phylogenetic trees to account for various selective forces affecting the long-term evolution of transposon loads in eukaryotic genomes [18]. In addition to full-length copies of transposons, there are truncated mobile elements, which may or may not utilize the transposition machinery [19,20]. As non-autonomous transposons utilize the expression products of autonomous elements for propagation and integration into genomes, predator–prey type models appear as a good tool to describe a specific interaction between them [21,22,23]. 

Xue and Goldenfeld introduced a stochastic individual-level model and its deterministic version to describe the dynamics of L1 and Alu, accounting for an interaction mechanism between these elements [23]. Their model predicted the noise-induced oscillations of the number of novel insertions of these transposons over several cell generations. Therefore, we can regard the transposon abundance at any instance in time as the oscillator state contained within a genome.

The potential deleterious effects induced by novel transposable-element insertions are arguably negligible in healthy humans, due to the mechanisms restricting transposon activity and ensuring low mobilization rates. Rough estimates for the transposition rate in the human population range from 1 out of 20 live births for Alu, to 1 out of 20–200 births for L1 [11]. However, these mechanisms may be significantly compromised in cancer. The disease state ensues a rise in the number of novel insertions at which the transposon activity becomes a dynamic variable in cancer genomes [7]. It may lead to genome size variations induced by the stresses a host cell experiences in disease, which makes it important to study the dynamical equilibrium between the host and the transposons in the context of the bioenergetic balance of a cell [24,25]. Furthermore, during their lifecycle, transposable elements may compete for the same pool of resources (energy, etc.) with other essential cellular processes, which makes the cellular environment an important player in the dynamics of retrotransposons. Here, we incorporate the competition for cellular resources into a previously proposed simple predator–prey model of the stochastic dynamics of the two autonomous and non-autonomous transposable elements, considering L1 and Alu as examples. Specifically, we show which dynamic effects may be expected when the transposons compete for ribosomes and energy.

## 2. Methods

### 2.1. Basic Predator–Prey Model of Transposon Dynamics

We began with a previously published, simple predator–prey model, describing the dynamics of L1 and Alu in a host genome, as represented by the following reactions [23]:

(1a) $$L\to L+R_{L},\;\;\mathrm{with}\;\mathrm{rate}\;v_{R},\;$$

(1b) $$R_{L}\to L,\;\;\mathrm{with}\;\mathrm{rate}\;b_{L}R_{L},\;$$

(1c) $$R_{L}+S\to 2S,\;\;\mathrm{with}\;\mathrm{rate}\;\left(b_{S}/V\right)R_{L}S,\;$$

(1d) $$R_{L}\to \emptyset ,\;\;\mathrm{with}\;\mathrm{rate}\;d_{R}R_{L},$$

(1e) $$L\to \emptyset ,\;\;\mathrm{with}\;\mathrm{rate}\;d_{L}L,$$

(1f) $$S\to \emptyset ,\;\;\mathrm{with}\;\mathrm{rate}\;d_{S}S.$$

Here, *L* and *S* stand for the number of active L1 and Alu transposon copies, respectively; *R_L_* represents the number of protein complexes encoded by L1 and used by *L* and *S* for integration into the genome; *v_R_* is the rate of *R_L_* production as *v_R_* = *b_R_L* in the basic model; *b_i_* and *d_i_* are rate constants. Equation (1a) describes the production of *R_L_* from each copy of active L1. *R_L_* consists of an endonuclease and reverse transcriptase; it binds the L1 mRNA from which it was translated and reverse-transcribed it into the genome. Therefore, a new *L* copy arises in accordance with the reaction (1b). Alu mRNAs are also able to bind to *R_L_* and to be retrotranscribed into the genome, as shown by reaction (1c), where *V* denotes the cell volume. Equation (1d–f) describe the degradation of protein *R_L_* and the silencing of the active transposons by shifting them to the untranscribed chromatin, or by other mechanisms.

Reactions (1) represent a stochastic version of the model in terms of copies of *L*, *S*, and a number of molecules of *R_L_*. Applying a mean field approximation to the stochastic model, the following deterministic version of the model can be obtained in the form of ordinary differential equations, which describe the dynamics of the mean concentrations $\bar{L}$, $\bar{S}$, and ${\bar{R}}_{L}$ [23]:

(2a) $$\frac{d\bar{L}}{dt}=b_{L}{\bar{R}}_{L}-d_{L}\bar{L},\;$$

(2b) $$\frac{d\bar{S}}{dt}=b_{S}\bar{S}{\bar{R}}_{L}-d_{S}\bar{S},\;$$

(2c) $$\frac{d{\bar{R}}_{L}}{dt}=v_{R}-b_{L}{\bar{R}}_{L}-b_{S}\bar{S}{\bar{R}}_{L}-d_{R}{\bar{R}}_{L},$$

where $v_{R}=b_{R}\bar{L}$ in the basic model.

**Figure 1:**
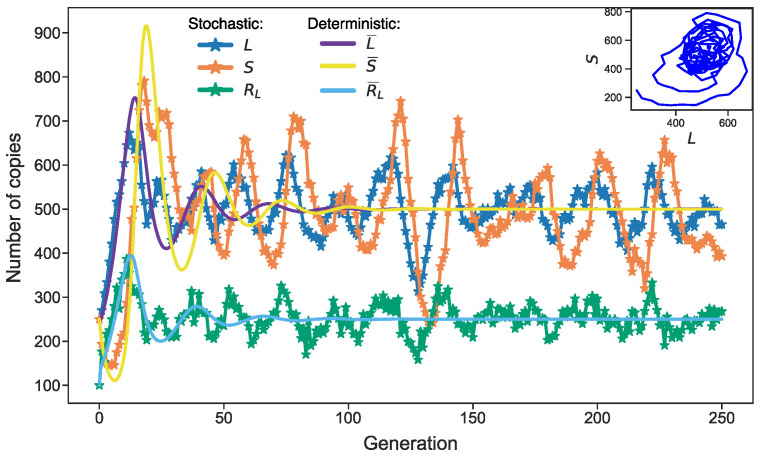
Dynamics of *L*, *S*, *R_L_*, and their mean values in the basic model. The parameter values are fixed as described in the Methods section. The stochastic dynamics were simulated with the Gillespie algorithm [26] applied to the reaction set (1), whereas the dynamics of the means were calculated by solving differential equations (Equation (2)). An approximate period of noise-induced oscillations equals 27 generations [23].

The competition between the L1 and Alu elements for protein complexes *R_L_* forms the basis of the predator–prey dynamics in the model. Figure 1 shows characteristic solutions of both the stochastic and deterministic models. The mean numbers of the aforementioned transposable elements and protein complexes *R_L_* converge to stationary values, which are stationary attractors in the space of variables *L*, *S*, and *R_L_*. The predator–prey nature of the deterministic model means that $\bar{L}$, $\bar{S}$, and ${\bar{R}}_{L}$ converge to this attractor in the form of damping oscillations. In mathematical terms, the eigenvalues of the Jacobian at the stationary state have non-zero imaginary parts in a linear stability analysis. Persistent stochastic deviations from the stationary state permanently keep the system in these oscillations. In contrast to the usual noise, these oscillations have a characteristic period encompassing many cell generations, with the parameters being determined by the imaginary parts of the eigenvalues [23]. Therefore, the minimal stochastic model (1) predicts the noise-induced oscillations of retrotransposon abundance in the genome. In what follows, we modify *v_R_* and rate constants in this model to account for retrotransposon dynamics under conditions of competition for ribosomes or energy.

### 2.2. Modification of the Model to Include Competition for Ribosomes

**Figure 2:**
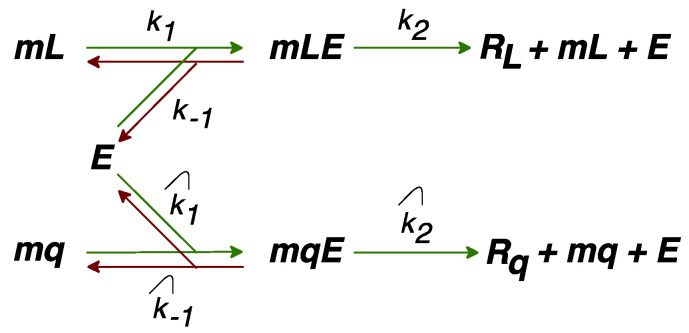
Kinetic scheme representing the competition for free ribosomes *E* between L1 mRNAs (*mL*) and all other translated RNAs in the cell (*mq*).

Reaction (1a) takes into account both the production of L1 mRNA, and its translation into a protein. The rate *v_R_* = *b_R_ L* (with constant *b_R_*) of this two-stage process implies a homogeneous influence from the cellular environment. To accommodate for the possible competition for free ribosomes between L1 mRNAs and other cellular RNAs, we modified the rate *v_R_* by temporarily introducing several new variables into the model: L1 mRNA (*mL*), mRNA of all other genes (*mq*), free ribosomes (*E*), mRNA–ribosome complexes (*mLE* and *mqE*), and proteins translated from *mq* (*R_q_*). Figure 2 presents the production of L1 proteins (*R_L_*) and all other cellular proteins (*R_q_*) in terms of these variables.

According to the scheme, the translation rate for protein *R_L_* equals *v_R_* = *k*_2_ *mLE*. We found *mLE* as a stationary solution to the following set of dynamical equations for the complexes shown in Figure 2:

$$\frac{dmLE}{dt}=k_{1}mL\cdot E-\left(k_{-1}+k_{2}\right)mLE,\;\frac{dmqE}{dt}={\hat{k}}_{1}mq\cdot E-\left({\hat{k}}_{-1}+{\hat{k}}_{2}\right)mqE.$$

The stationary solution for *mLE* has the form:

$$mLE=E_{\mathrm{tot}}\frac{mL}{c_{1}K_{d}+mL},$$

where *E*_tot_ = *E* + *mLE* + *mqE* is the total concentration of ribosomes, $K_{d}=\left(k_{2}+k_{-1}\right)/k_{1}\approx k_{-1}/k_{1}$ is the equilibrium dissociation constant associated with the *mLE* complex formation, $c_{1}=1+mq/{\hat{K}}_{d}$, ${\hat{K}}_{d}=\left({\hat{k}}_{2}+{\hat{k}}_{-1}\right)/{\hat{k}}_{1}\approx {\hat{k}}_{-1}/{\hat{k}}_{1}$ is the equilibrium dissociation constant for the *mqE* complex formation, and *mq* in $c_{1}$ is assumed to be a stationary concentration of all transcribed RNAs (other than transposon RNAs) in the cell.

Allowing a linear relationship between the numbers of L1 mRNAs and genomic copies of transposons, we introduced a linear approximation constant *c*_2_ (*mL* = *c*_2_*L*) and obtained the final expression for the rate *v_R_* in the modified model:

(3a) $$v_{R}=\nu_{\max}\frac{L}{k_{r}+L},\;\;\nu_{\max}=k_{2}E_{\mathrm{tot}},$$

(3b) $$k_{r}=\frac{c_{1}}{c_{2}}K_{d}=\frac{K_{d}}{c_{2}}\left(1+\frac{mq}{{\hat{K}}_{d}}\right),$$

where *v*_max_ is the maximal translation rate for *R_L_*, and *k_r_* is a constant representing the strength of competition for ribosomes (‘competition parameter’). We used this *v_R_* as a new reaction rate (see Equation (1a)) in the stochastic simulations. Likewise, when simulating the dynamics of the mean concentrations, we used the new *v_R_* in Equation (2c), but with *L* replaced by $\bar{L}$. We analyzed the influence of competition for ribosomes by simulating the dynamics for various values of the competition parameter *k_r_*.

### 2.3. Modification of the Model to Include Competition for Energy

The competition of transposons for cellular energetic resources is acknowledged in the model (1)–(2), by adding energy dependence into the rate constants *b_R_*, *b_L_*, and *b_S_*, in accordance with the Michaelis–Menten kinetics. We introduced the dependence on a new parameter *e* that quantified the available energy in the cell, expressed, for example, as the amount of ATP molecules, as follows:

(4a) $${\tilde{b}}_{R}=b_{R}\frac{e}{k_{\mathrm{tr}}+e},\;$$

(4b) $${\tilde{b}}_{L}=b_{L}\frac{e}{k_{\mathrm{int}}+e},$$

(4c) $${\tilde{b}}_{S}=b_{S}\frac{e}{k_{\mathrm{int}}+e}.\;$$

Here, the additional parameters *k*_tr_ and *k*_int_ stand for energy consumption of translation and transposon integration processes, respectively. We used the new constants ${\tilde{b}}_{i}$ instead of *b_i_* in (1)–(2), and analyzed the influence of energy dependence by simulating the dynamics in the model for various values of *e*. In this model, we ignored the competition for ribosomes and thus set $v_{R}={\tilde{b}}_{R}L$.

We also considered a mixed model, which combined both types of competition for cellular resources, i.e., ribosomes and energy. In this model, we used *v_R_* from (3) scaled by *e*/(*k*_tr_ + *e*) for the translation rate and (4b)–(4c) as the integration rate constants.

### 2.4. Parameter Values

We fixed the parameter values from the basic model according to ref. [23]: *b_R_* = 2, *b_L_* = 1, *b_S_* = 1, *d_R_* = 2, *d_L_* = 0.5, *d_S_* = 0.5, and *V* = 500. These values provided the characteristic predator–prey dynamics of transposon replication and can be used as reference values for further modifications.

To estimate *v*_max_, we have:

$$\nu_{\max}=k_{2}E_{\mathrm{tot}},$$

where *E*_tot_ is the total concentration of ribosomes, and *k*_2_ is the translation rate constant. Assuming the translation rate equals 5 amino acids (aa) per second (BNID 104598 [27]) and length of L1 mRNA equals 2000 aa [28], we get the following translation speed per one protein:

$$k_{2}=\frac{5\;\mathrm{aa}/\sec}{2000\;\mathrm{aa}}=0.0025\;\sec^{-1}.$$

Taking the estimate *E*_tot_ = 50,000 ribosomes for *E. coli* (BNID 108600 [27]), we get:

$$\nu_{\max}=0.0025\times 50,000=125\;\mathrm{molecules}\;\mathrm{per}\;\sec.$$

To translate seconds to cell generations, we estimated the generation turnover in *E. coli* to be 20 min (BNID 103514 [27]). Finally, converting molecules to concentration units, we divided by the chosen volume *V* = 500. This value can be considered as an estimate for the *E. coli* volume in cubic microdecimeters (BNID 114925 [27]). Thus, we have:

$$\nu_{\max}=\frac{125\;\frac{\mathrm{molec}.}{\sec.}\times 1200\frac{\sec.}{\mathrm{gen}.}}{500µ{\mathrm{dm}}^{3}}=300\;\frac{\mathrm{molec}.}{µ{\mathrm{dm}}^{3}\times \mathrm{gen}.}$$

Given this value of *v*_max_, we took the value *k_r_* = 149 as a reference for *k_r_* (‘normal conditions’ in terms of the competition for ribosomes), since the modified model with such *v_R_* and *k_r_* provided the same dynamics as the basic model (Figure 1).

We chose parameters *k*_tr_ and *k*_int_ (e.g., *k*_tr_ = 0.2 and *k*_int_ = 3.2) to maintain the previously reported ratio between the Michaelis–Menten parameters with respect to translation and integration rates [29,30]. The specific values are not important, since the energy parameter *e* has arbitrary units.

### 2.5. Stability Analysis

We calculated the stationary solutions of the models (Equation (2)), modified as described above, and analyzed their linear stability against a range of values for the competition parameter *k_r_*, and the energy level *e*. Stable stationary solutions attracted the dynamical solutions, starting from various initial conditions. As *S* may have become zero for some values of *k_r_* in the process of stochastic simulations, we also investigated the stability of the stationary solutions for the following equations. These equations represent the modified model (2) where the equation for *S* is excluded and *S* is set equal to zero in the other equations:

(5a) $$\frac{d\bar{L}}{dt}=b_{L}{\bar{R}}_{L}-d_{L}\bar{L},\;$$

(5b) $$\frac{d{\bar{R}}_{L}}{dt}=\nu_{\max}\frac{\bar{L}}{k_{r}+\bar{L}}-b_{L}{\bar{R}}_{L}-d_{R}{\bar{R}}_{L}.\;$$

### 2.6. Stochastic Simulation in Cell Population

To simulate the stochastic dynamics in the models we used the Gillespie algorithm [26]. We ran simulations 3000 times for the same parameter values and initial conditions and interpreted the results as the stochastic dynamics in a population of 3000 cells. The initial conditions in each cell were as follows: *L* = *S* = 250 copy numbers per cell, and *R_L_* = 100 copy numbers per cell. 

To minimize the influence of the initial conditions, we considered the first 100 generations in these simulations as a buffer period and only analyzed the dynamics afterwards. Since *L* and *S* could drop to zero due to stochastic events, we excluded cells that lost both active transposons in the buffer period from further analysis. Therefore, beyond the buffer period, we observed a population of cells with at least one active transposon in each cell, and defined the 101st generation as the first one of interest for further downstream analysis.

We characterized the possible noise-induced deaths of either *S* or *L* during the stochastic simulation in the population using the Kaplan–Meier survival functions for *L* and *S*, defined as follows [31]:

(6) $$F\left(i\right)=\underset{i}{\prod }\left(1-\frac{d_{i}}{n_{i-1}}\right),\;$$

where *d_i_* is the number of ‘deaths’ of *L* or *S* (i.e., the number of cells where *L* or *S* became zero) that occurred between cell generations i−1 and *i*. *n_i-1_* is the number of cells with *L* or *S* that ‘survived’ (i.e., the number of cells with non-zero *L* or *S*) by generation i−1. The product in (6) was computed over all generations *i* in the stochastic simulation; this survival function can be interpreted as the probability that active transposons (either *L* or *S*) remained in the cell by *i*th generation at the given competition level.

## 3. Results

### 3.1. Model of Transposon Dynamics Under Competition for Free Ribosomes

The set of reactions (1) from the basic model by Xue and Goldenfeld are minimal individual-level representations of the specific interaction between the autonomous (L1) and non-autonomous (Alu) retrotransposons. The values of the corresponding rate constants orchestrated the transcription and translation processes. Another simplification concerns the fact that the numbers of copies of L1 (*L*), Alu (*S*), and the protein coded by L1 (*R_L_*) stochastically evolved within the cellular environment that remained unperturbed. In other words, the constant parameters represented and accumulated the joint influence of both the other cellular processes and the molecular factors.

The competition for free ribosomes should effectively reduce the synthesis rate of *R_L_* (*v_R_* = *b_R_L* in the basic model), so we modified the model and derived a new form of this rate (Equation (3) in the Methods section), which accounted for the influence from the other RNAs in the cell under the assumption of a quasi-stationary approximation for the kinetic scheme shown in Figure 2. The new parameter *k_r_* in the modified model stands for the competition for free ribosomes between mRNAs encoded by L1 and other genes, by means of four factors. According to (3b), a higher competition impact on transposons occurred at higher values of *k_r_*, and this happened if (a) the number of competing non-transposon RNAs was large, or (b) their affinity to ribosomes was high, or (c) the transcription rate for L1 was small, or (d) the affinity of L1 mRNAs to ribosomes was low. The total number of L1 and the maximal translation rate were not associated with the competition for ribosomes, but did have an impact on the translation rate for *R_L_*.

We found a value of *k_r_* (*k_r_* = 149), for which the modified model with *v_R_* from Equation (3) provided the same dynamics as the basic model in Figure 1. Therefore, we regarded both the dynamics and the value of competition parameter *k_r_* as standard conditions, and investigated how the dynamics changed and what new effects appeared as competition grew.

### 3.2. Stability Analysis Predicts Two Different Attractors for L1

**Figure 3:**
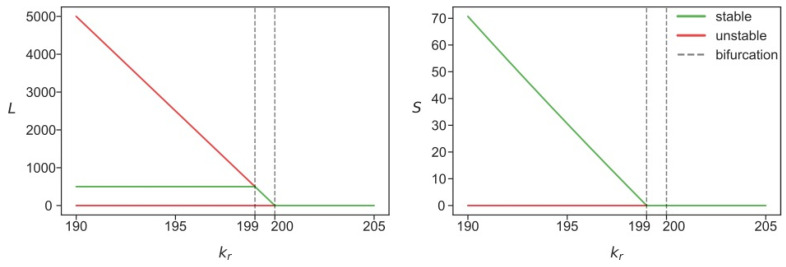
Stationary solutions for *L* and *S* (expressed as numbers of copies per cell) in the deterministic model (2)–(3), as functions of the competition parameter *k_r_*. Red and green colors differentiate between linearly unstable and stable solutions, respectively. The unstable branch *S* = 0 in the right panel corresponds to both unstable branches in the left panel.

There were three stationary solutions in the deterministic version of the modified model at *k_r_* with values smaller than the value of *k_r_* = 199. Of these, only one solution was stable (Figure 3). The stationary *S* of the only stable solution branch decreased as the competition strength grew, whereas the stationary *L* remained insensitive to the parameter within the aforementioned range of its values. At the first bifurcation point (*k_r_* = 199), *S* dropped to zero and *L* started to decrease. At the second bifurcation point (*k_r_* = 200), the stationary level of *L* decreased to zero as well, indicating that both transposons stopped replicating, either at this level or at the higher levels of competition in the steady state. 

This picture, however, only partially describes what happens in the stochastic system. Since the stationary solution with zero *S* was unstable for *k_r_* < 199, it follows that, under the propositions of the deterministic model and given these competition levels, the process starting at any non-zero *S* could not reach a state with zero *S*. However, *S* became relatively small close to the bifurcation point (Figure 3), and stochastic effects may have changed the dynamics predicted by the deterministic solution. In particular, a stochastic simulation may have encountered a series of degradation reactions in the case of Alu elements, resulting in a state with *S* = 0 at some point in time. This situation corresponds to the state of absence of active Alu retrotransposons in the genome and, consequently, to a stop in replication for this particular transposon. The reduced deterministic model obtained by excluding *S* from the equations (Equation (5) in Methods) describes the deterministic dynamics corresponding to exactly this particular case.

**Figure 4:**
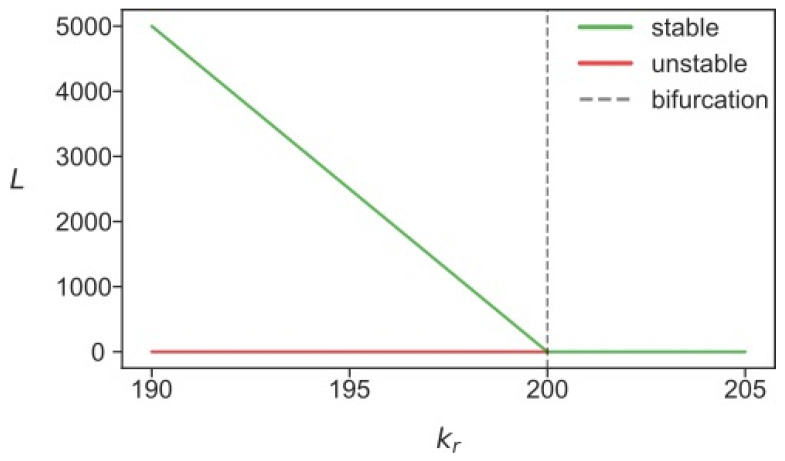
Stationary solutions for *L* in the reduced deterministic model (5) as functions of the competition parameter *k_r_*.

Figure 4 shows the stationary solutions for *L* in the reduced model. In contrast to Figure 3, the solution with non-zero *L* became stable at *k_r_* values less than 200. This stationary solution attracted all dynamical solutions with zero *S*. Since the absence of *S* effectively removed the constraints imposed on the dynamics of *L*, this attractor yielded a higher level of *L* than the attractor presented as the green line in Figure 3. Therefore, any noise-induced decrease of *S* to zero should result in the dynamical increase in *L* to the higher mean levels. If, in a likewise fashion, *L* became zero due to noise effects or due to high values of *k_r_* (see the bifurcation diagram in Figure 3), replication of both *L* and *S* would stop in the cell, hence, the state *L* = *S* = 0 permanently sets in the cell.

Thus, the stability analysis revealed two possible stationary regimes for L1 transposons, depending on whether Alus were present or extinguished in the cell, due to the competition for ribosomes between L1 RNAs and other RNAs. The L1 copy number tended over time towards a lower steady-state value if Alu was present, and to a higher steady-state level otherwise. Within this picture, the cell also could be in a state characterized by an intermediate *L* fitting between the two steady-state values. This happens when Alu has only recently stopped replicating in this cell and the stationary limit has not yet been reached.

### 3.3. Stochastic Effects in Transposon Dynamics Divide Cell Population into Pools of Cells with Essentially Different Numbers of L1

We demonstrated the existence of two regimes for L1 by stochastically simulating the transposon dynamics in a cell population, under various competition levels for ribosomes. As *k_r_* approached the bifurcation value, cells in the simulation divided into three pools, depending on the number or L1 and Alu elements. The first pool included cells with non-zero *S* and *L*, in which the predator–prey dynamics continued. The second pool contained cells in which *L* was non-zero and *S* became zero due to stochastic effects, i.e., no active Alu elements. The third pool included cells with the state *L* = *S* = 0; the replication of retrotransposons stopped completely. The pool sizes depended on *k_r_* and simulation time. 

**Figure 5:**
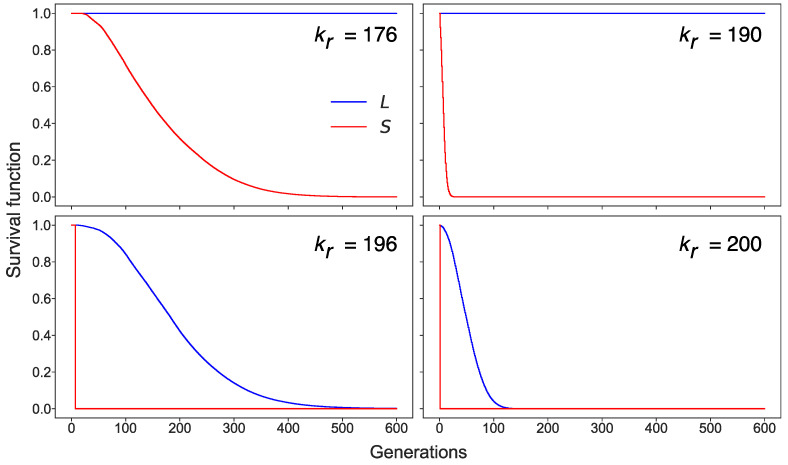
Dynamics of the survival function from (6) for *L* (blue) and *S* (red) and various values of the competition parameter *k_r_*, calculated from a stochastic simulation of transposon replication in a population of 3000 cells. The survival function of *S* shows the proportion of cells belonging to the first pool, and the difference between survival functions of *L* and *S* indicates the proportion of cells from the second pool.

To visualize how the pools evolved, we calculated the Kaplan–Meier survival functions of *L* and *S* in the cell population, which can be interpreted as the probability that the active transposons (either *L* or *S*) remained in the cell by the *i*th cell generation at the given competition level (Figure 5). The survival dynamics showed that cells with non-zero *S* disappeared rapidly with time. The sharp drop happened long before the bifurcation value of the competition parameter. Remarkably, the L1 elements remained active in these cells. The number of cells with non-zero *L* started to decrease with time only when the competition strength came close to the bifurcation value.

**Figure 6:**
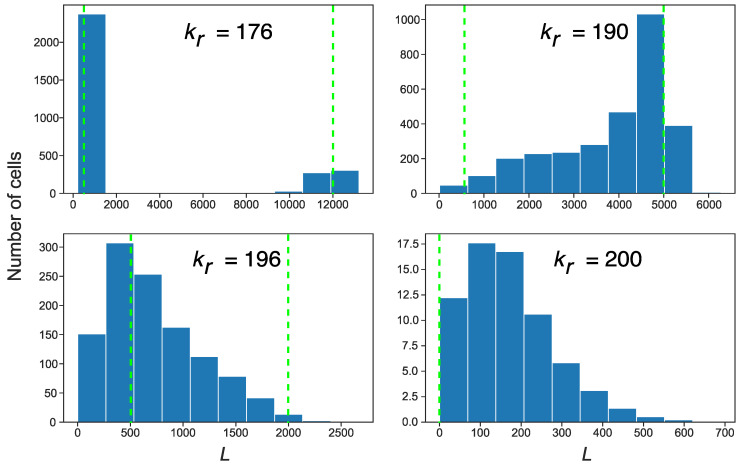
Distribution of *L* over cells with active transposons (cells with non-zero *L*) in the population of 3000 cells, for various values of the competition parameter. The histograms show values of *L* averaged over 300 generations in the stochastic simulation. Vertical dashed lines show the stable stationary solutions predicted by the full deterministic model (left line) and reduced one (right line); for *k_r_* = 200, both solutions give *L* = 0 (see Figure 3 and Figure 4).

It is noteworthy that the two stationary solutions with non-zero *L,* described above, acted as two quasi-attractors in the stochastic model of the cell population, each in its pool of cells. The stable solution from Figure 3 governs the dynamics in cells with non-zero *S* (first pool), whereas the solution from Figure 4 corresponds to the cells with zero *S* (second pool). To find out which attractor was dominant in the cells depending on the different levels of competition for ribosomes, we calculated the distributions of the time-averaged *L* in the population for various competition levels (Figure 6). These distributions demonstrated a qualitative difference. In the case of competition levels far from the bifurcation value of *k_r_* (*k_r_* = 176 in Figure 6), *S* remained non-zero for the majority of cells (i.e., these cells belong to the first pool), with *L* being close to the stationary value obtained from the predator–prey dynamics. However, the fluctuations in the value of *S* in a small number of cells led to the inactivation of the Alu elements. Thus, a small portion of the population formed the second pool of cells, characterized by a larger stationary *L*. For stronger competition (*k_r_* = 190 in Figure 6), the aforementioned pool dominated the cells, and thus the second attractor became the main mode in the distribution. The long tail of the distribution encompassed the cells with the intermediate dynamics of growing average *L*. If *k_r_* increased further (*k_r_* = 196 in Figure 6), an essential number of cells lost both *L* and *S* and, consequently, very few cells appeared in the second pool. Finally, at the second bifurcation value of the competition parameter (*k_r_* = 200), the stationary attractor took zero value for both *L* and *S*. This meant that only a few cells with active transposons showed intermediate fluctuations in their number, which eventually subsided.

**Figure 7:**
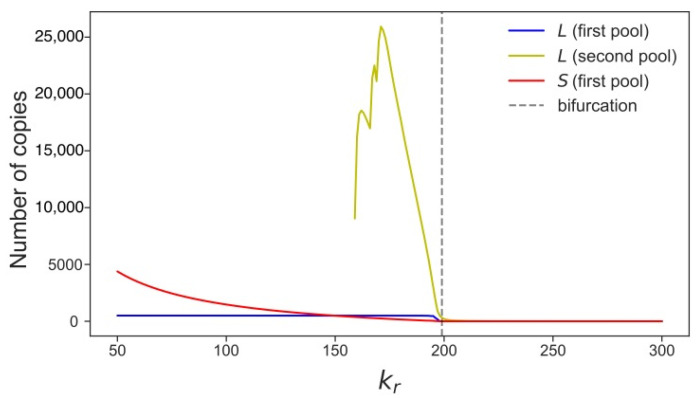
The number of retrotransposon copies, averaged over 600 generations of stochastic simulation in a population of 3000 cells, as a function of the competition parameter. The curves are interpolations between the values obtained from calculations. The straight line in the curve for *L* in the second pool corresponds to the stable stationary solution shown in Figure 4. The other part of this curve represents cells with the intermediate dynamics of *L*. The curves for *L* and *S* in the first pool correspond to the stable stationary solution presented in Figure 3.

Figure 6 shows how an increase in the competition parameter qualitatively changes the distribution of *L* in a cell population, from bimodal to various skewed forms of unimodal. These distributions bear the imprint of different transposon dynamics in different pools of cells, such as the dynamics governed by distinct attractors for *L*. These distinct attractors can be captured in stochastic simulations by averaging *L* and *S* in different pools of cells (Figure 7). The noise-induced disappearance of *S* started approximately at *k_r_* > 150, and cells with the larger *L* started to form the second pool. 

### 3.4. Rising Competition for Ribosomes Affects Noise Levels and Oscillation Parameters of L and S

**Figure 8:**
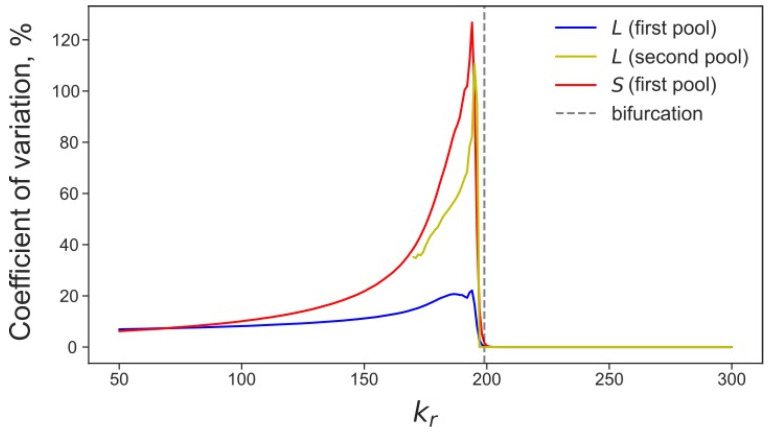
Coefficient of variation of *L* and *S* (ratio of the standard deviation to the mean) calculated over 600 generations of stochastic simulation in a population of 3000 cells and averaged in different pools, for various values of the competition parameter.

**Figure 9:**
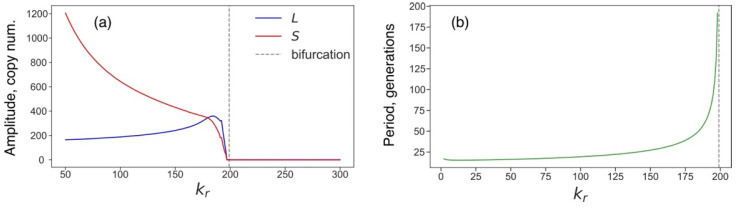
Characteristic parameters of noise-induced predator–prey oscillations of *L* and *S* for various values of the competition parameter. (**a**) Time-averaged amplitudes of *L* and *S* oscillations in the cells from the first pool estimated from the stochastic simulation in a population of 3000 cells. The amplitude is the difference between the maximum and minimum of *L* or *S* found in a frame length of 100 generations and repeated by moving the frame with 10-generation increments within the 600-generation interval. (**b**) Period of the predator–prey oscillations in the first pool.

The L1 and Alu copy numbers demonstrated noise-induced oscillations with a specific amplitude and period under normal conditions (Figure 1). We can also use the ratio of the standard deviation to the mean to characterize the noise level of stochastic systems. We found that all these parameters significantly changed as the competition for ribosomes grew. The noise in the numbers of L1 and Alu increased in all pools of cells with the growth of the competition parameter. This effect was most pronounced for *S* in the first pool and *L* in the second pool (Figure 8). The oscillation parameters of *L* and *S* demonstrated qualitatively different behavior (Figure 9); the oscillation amplitude of *S* decreased as the competition rose. This tendency corresponded to a decreasing average *S*. At the same time, oscillations of *L* demonstrated a local increase in the amplitude preceding the critical value of the competition parameter (Figure 9a). The oscillation period increased for both transposons, as the competition parameter approached the critical value (Figure 9b).

### 3.5. Transposon Dynamics under Competition for Energy

**Figure 10:**
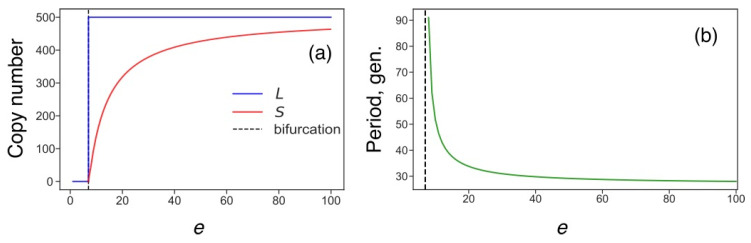
Energy dependence of stationary solutions and oscillation period. (**a**) Stable stationary solution for various values of the energy parameter (*e*, arbitrary units). (**b**) Period of noise-induced predator-prey oscillations of *L* and *S* for various values of the energy parameter.

We investigated the competition for energy in the new version of the model by varying *e* in the new rate constants from (4) and neglecting the competition for ribosomes. Smaller levels of *e* correspond to lower energy resources in the cell and, therefore, a higher competition level. The stability analysis in the deterministic model showed that there was only one stable stationary solution, which varied with *e* very slowly and decreased sharply if, and only if, *e* was close to the bifurcation value (Figure 10a). For *e* smaller than the bifurcation value (*e* = 7.3), no transposon replication was possible. Similar to the case of competition for ribosomes, the period of noise-induced fluctuations in the predator–prey dynamics of *L* and *S* increased as the energy became less accessible (Figure 10b). The stochastic simulations showed that the average amplitude of the *L* oscillations also grew when approaching the bifurcation level. Therefore, the competition for energy led to qualitatively similar effects in the transposon dynamics as were observed in the case of competition for ribosomes.

**Figure 11:**
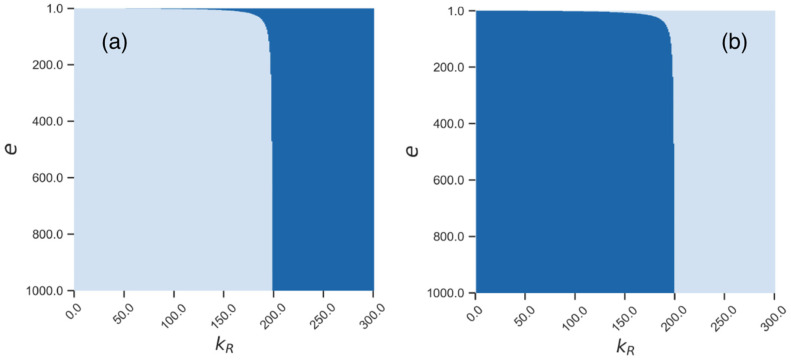
Stability of different stationary solutions in the space of parameters *kr* and *e*. Light blue shows stable zones, and dark blue shows unstable zones for (**a**) the non-zero stationary solution from Figure 3 and (**b**) the zero stationary solution.

We also investigated the mixed model, which combined both types of competition for cellular resources (ribosomes and energy). The overall behavior of the mixed model was qualitatively similar to those described above; however, the described effects were shifted to smaller values of the competition parameter *k_r_* as the parameter *e* decreased. Figure 11 illustrates this fact, as the critical value of *k_r_*, that delimits the existence of the non-zero stationary solution in the deterministic model, slowly decreases with the decreasing *e*.

## 4. Discussion

**Figure 12:**
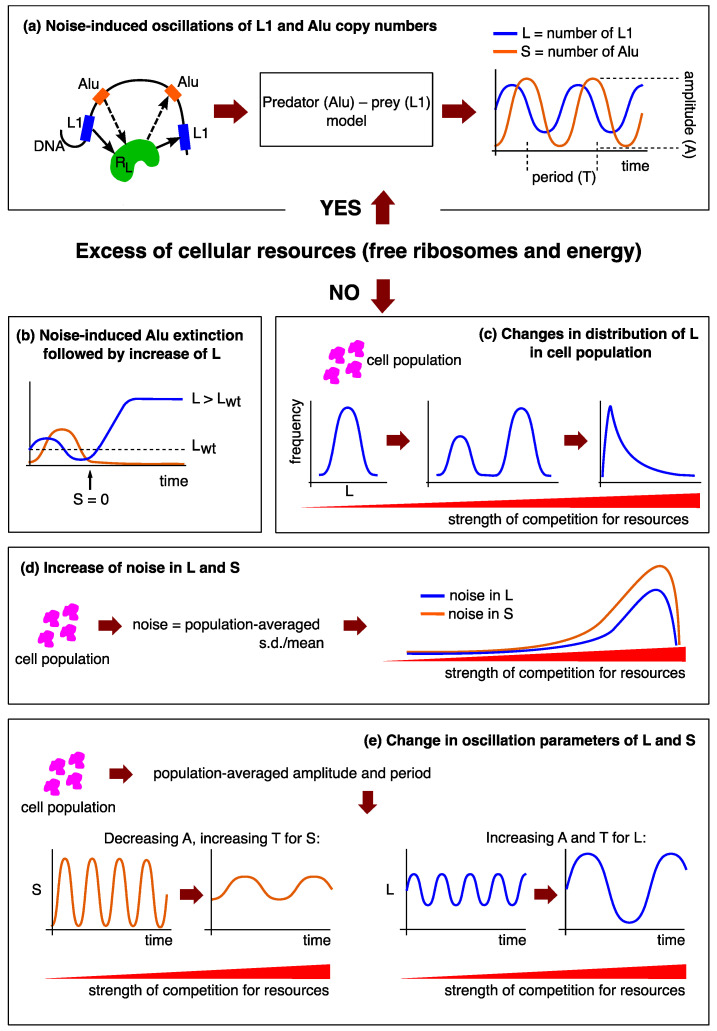
Summary of the results obtained in the study. (**a**) When there is no competition for cellular resources within the cell, the previously published predator–prey model predicts the noise-induced oscillations of *L* and *S* in the genome, with specific amplitude *A* and period *T*. Solid arrows label the production path of *R_L_* and its usage as reverse transcriptase, whereas the dashed arrows designate that Alu competes with L1 for this molecule to insert into genome [23]. Stochastic effects in transposon dynamics if L1 and Alu compete for cellular resources: (**b**) Under pressure of competition for resources, Alu elements may become inactivated, leading to larger L1 abundance. *L*_wt_, time-averaged *L* in the absence of competition for resources. (**c**) Competition for resources transforms the population distribution of *L*, and a bimodal distribution appears at some competition levels because of the effect from (**b**). (**d**) The L1 and Alu copy numbers become more variable as approaching the complete inactivation. (**e**) Competition for resources transforms the amplitude *A* and period *T* of the predator–prey oscillations exhibited by the L1 and Alu copy numbers.

We investigated a simple model that simulated the predator–prey dynamics of transposon replication under demanding conditions: competing with the other cellular processes for ribosomes and energy. Our results clearly indicated that the replication dynamics acquired several characteristic properties when the competition reached substantial levels (Figure 12). Firstly, noise-induced (i.e., by stochastic fluctuations in the system) deactivation of Alu elements became possible, which triggered an increase in L1 numbers (Figure 12b). Secondly, the cell population demonstrated a bimodal distribution of L1 elements; this distribution significantly changed with the increasing competition, as (a) noise-induced inactivation of both transposons occurred in a larger number of cells and, (b) more cells manifested the intermediate dynamics switching between the two possible L1 levels (Figure 12c). Third, L1 and Alu copy numbers became noisier in the population, that is, the retrotransposon abundance in the host genome varied more significantly across cells and time (Figure 12d). Fourth, the noise-induced predator–prey oscillations decreased in amplitude for Alu elements, but increased for L1 retrotransposons, whereas the period of these oscillations slowly increased with the competition strength for both types of transposons (Figure 12e). Finally, the replication process completely stopped at a critical level of competition.

The competition for cellular resources between different cellular agents may be considered in the context of bioenergetic costs spent on basic internal processes [24,25,29,32]. These costs are subject to the evolutionary tuning of cellular processes leading to their homeostasis. Various stressful conditions, such as diseases, alter gene expression, metabolic and signaling pathways and can lead to the redistribution of resources [33]. A change in the bioenergetic balance associated with this shift from homeostasis may result in various unexpected intracellular phenomena, which may be harmful to the organism or, on the contrary, may bring new adaptive opportunities. All the dynamical features of transposon replication described in our work can be considered as dynamical markers for possible stress conditions in a cell population. 

A malignant transformation may induce various stress conditions associated with an increasing competition for energy. Cancer cells shift their metabolism to the conditions of limited energy (Warburg effect), so we may expect the reshaped energy demands to affect both transcription and translation [33,34,35]. This expectation correlates with the results of multiple studies showing that cells can be very sensitive to the instant energy levels and initiate cell death programs if these levels are low [36,37,38]. Even a moderate (three-fold) drop in ATP concentrations may lead to apoptosis or necrosis [38]. This renders it plausible that transposons compete with other cellular agents for the decreasing ATP pool of cancer cells, especially given the higher rates of transposon insertion in cancer [6,7]. In this case, we can consider the stochastic effects described in our study as a novel manifestation of existing cancer hallmarks; however, whether this competition is real in the case of retrotransposable elements undoubtedly requires solid experimental verification.

Our results, observed in the case of competition for ribosomes, complement other studies that consider the finite pool of available ribosomes as a rate-limiting factor [39,40,41]. Such competition induces cooperative behavior between different types of mRNA molecules, as modifications in the translation rates of a specific mRNA molecule yield a change in the translation rates of all other mRNA molecules [39]. A combination of modeling and experiments have revealed evidence for similar ties between the production rates of proteins, without an apparent regulatory path between them, thus providing evidence of competition for translational resources [40]. Other models describe the balance between the burden that a composition of the transcript pool exerts on the translation machinery and the ribosomal supply [41]. We presume that the increase in the noise of L1 and Alu numbers reported in our study may also affect the pool of other transcripts in the cell, through interactions mediated by the competition for the pool of free ribosomes.

Interestingly, viruses are another factor that can promote cell transformation associated with the type of stress that activates or enhances competition for resources. After infection, the viral mRNAs compete with the host for ribosomes and eukaryotic initiation factors, recruiting ribosomes to viral and cellular mRNAs [42,43]. For example, the virulence factor SARS-CoV-2 NSP1 competes with RNAs for binding the 40S ribosome subunit, thus inhibiting the protein synthesis in human cells [44]. We may expect the higher competition level experienced in infected cells to launch the dynamical effects that we have reported for transposons.

Our results provide good reason to speculate on the possibility of detecting a cell in a stress state, using a high level of internal competition for resources as a marker, manifested by measured numbers (or ratios) of L1 and Alu elements in the genome. From this point of view, all effects presented in Figure 12 are hypothetically detectable, if we count *L* and *S* in the data obtained by the bulk sequencing of cells. We can infer the dynamics of all the parameters from these estimates, and make conclusions if the transposons participate in the competition.

The results presented here are purely theoretical, as our computational experiment aimed to find and investigate the details of potential mechanisms associated with an increase in the competition for resources. In particular, we fixed most of the parameter values associated with retrotransposon replication from previous works [23], with our main purpose being to reproduce the predicted predator–prey dynamics, and estimate the remainder, considering various numbers reported for the processes involved [27]. This limits the interpretation of the results and their relation to specific organisms or cell types, based on their homeostatic parameters. However, as mentioned above, the results may facilitate situations in which specific values of replication parameters are transient [7,45]. Therefore, it is important to characterize the theoretical possibilities allowed by the nonlinear nature of the replication dynamics, and our results should be informative in this context.

The presented model describes the transposon replication process in an individual cell, but some of the reported effects are best exhibited and quantified in a population of cells. This is a consequence of the stochastic nature of these effects, such as the noise-induced extinction of active Alu or L1, which happens with a certain probability. The predator–prey dynamics of these transposons is primarily a stochastic feature of each cell in the population [23]. Therefore, by introducing the competition for cellular resources into the model, we translated the stochastic effects from the level of individual cells to the level of cell populations. In this respect, our modeling approach connects the single cell and population-level models of transposon activity [20]. In the future, we hope to incorporate more details into the model and explore the biologically plausible dynamical scenarios for specific organisms or cell types.

The model can be generalized by extending the number of ‘players’, as well as by including other types of competition for resources, thus opening future intriguing research avenues.

**Figure 13:**
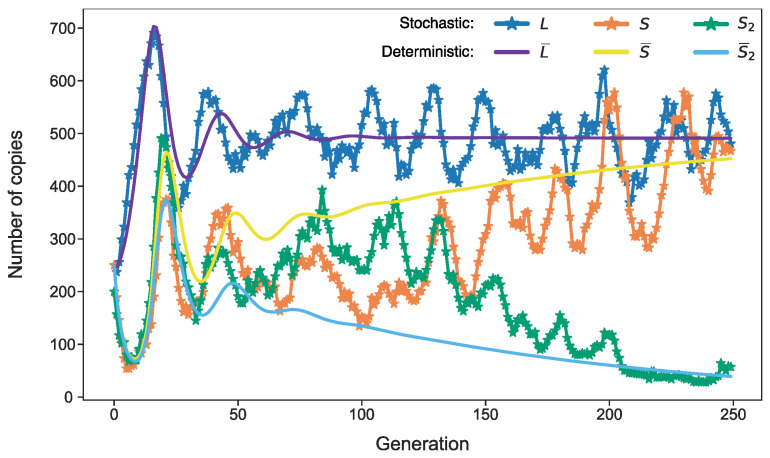
The same dynamics as presented in Figure 1, but for a model with one additional predator *S*_2_. This model has additional reactions and equation for *S*_2_ in (1)–(2), which coincide with the reactions and equation for *S*. We assume that *S*_2_ has the same parameters as *S*, except *b_S2_* = 0.98*b_S_. In case *b_S_* < *b_S_*_2_, *S* asymptotically vanishes and *S*_2_ remains as the only predator (not shown). Dynamics of *R_L_* is not shown.

Specifically, L1 can compete for the protein machinery essential for propagation not only with Alu, but also with another non-autonomous retrotransposon SVA [46]. This introduces a second ‘predator’ (*S*_2_) into the model, which participates in the noise-induced oscillations. Naturally, the long-term behavior of these oscillations generally depends on the parameter values. In our case, a small difference in the insertion rate constants of the two non-autonomous retrotransposons resulted in the eventual inactivation of one of them (Figure 13). The model can be extended further by accounting for the competitive binding of *S* and *S*_2_ to *R_L_*.

Some mRNAs utilize the enzymatic activity of L1 to reverse transcribe into DNA, and in the process generate genomic copies called processed pseudogenes. This biological phenomenon creates further questions and offers further opportunities for model extension, as some processed pseudogenes are a byproduct of LINE1 mobilization [47,48]. Accounting for these events under the active competition between L1 transcripts and non-transposon mRNAs for ribosomes will ensue, accounting for pseudogene-related mRNAs separately from other non-transposon mRNAs. The latter competes with L1 transcripts only for ribosomes, whereas the former plays the additional role of the second predator (analog of *S*_2_ described above), thus participating in two types of competition simultaneously. 

The environment, including various internal resources, is of paramount importance for upkeeping the homeostatic state of the cell. This study sought answers to the questions that remain to be addressed in focused wet lab experiments, concerning the understudied details of the interplay between genomic parasites and other cellular processes consuming energy and resources. In their absence, our models facilitate the development of new approaches for the evaluation of cell states in normal conditions and in disease.

## Data Availability

Not applicable.
